# Clinical and Radiographic Outcomes of Total Elbow Arthroplasty Using a Semi‐constrained Prosthesis with a Triceps‐preserving Approach over a Minimum Follow‐up Period of 4 Years

**DOI:** 10.1111/os.13698

**Published:** 2023-04-19

**Authors:** Qing Zhang, Ming Xiang, Jin‐song Yang, Fei Dai

**Affiliations:** ^1^ Department of Upper Limb Sichuan Provincial Orthpaedics Hospital Chengdu China

**Keywords:** Distal humerus fracture, Joint prosthesis, Osteoporosis, Total elbow arthroplasty

## Abstract

**Objective:**

Complications related to triceps after total elbow arthroplasty (TEA) have become a major surgical concern. The triceps‐preserving approach has the advantage of not disturbing the insertion of triceps but is disadvantaged by the reduced exposure of the elbow joint. The aim of this study was to investigate the clinical and radiological outcomes of TEA with a triceps‐preserving approach and to compare the outcomes of TEA to treat arthropathy with that of TEA to treat acute distal humerus fracture.

**Methods:**

From January 2010 to December 2018, 23 patients undergoing primary TEAs were retrospectively reviewed with a mean follow‐up time of 92.6 months (range, 52–136 months). Each TEA was performed using the triceps‐preserving approach with a semi‐constrained Coonrad–Morrey prosthesis. Patient demographics, range of motion (ROM), pain visual analogue scale (VAS), and triceps strength (Medical Research Council [MRC] scale) were compared before and after surgery. The Mayo Elbow Performance Score (MEPS), Disabilities of the Arm, Shoulder, and Hand (DASH) score, radiographic outcome, and complications were evaluated at follow‐up.

**Results:**

In total, seven males and 16 females were included in this study, with a mean age of 66.1 years (range:46–85 years). By the last follow‐up, pain had been significantly relieved in all patients. The average MEPS in the arthropathy group and fracture group were 90.8 ± 10.3 points (range: 68–98 points) and 91.7 ± 0.4 (range: 76–100 points), respectively. The average DASH of the arthropathy group and fracture group was 37.3 ± 18.8 points (range: 18–52 points) and 38.4 ± 20.1 (range: 16–60 points). At the last follow‐up after surgery, the mean flexion arcs in the arthropathy group and fracture group were 100.4° ± 24.1° and 97.8° ± 28.1°, respectively. The mean pro‐supination arcs in the arthropathy group and fracture group were 142.4° ± 15.2° and 139.2° ± 17.5°, respectively. There were no significant differences in clinical outcomes between the two groups (*P* ≥ 0.05). Triceps strength was normal (MRC grade V) in 15 elbows and good in eight elbows. None of the cases experienced weakness of the triceps strength, infection, periprosthetic fractures, or prosthesis breakage.

**Conclusions:**

The clinical and radiographical outcomes of TEA with the triceps‐preserving approach were satisfactory in patients with distal humerus fracture, osteoarthritis and rheumatoid arthritis.

## Graphical abstract

Clinical and radiographic outcomes of total elbow arthroplasty with triceps‐preserving approach: with minimal 4‐years follow‐up.

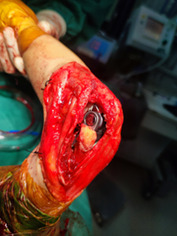

## Introduction

Total elbow arthroplasty (TEA) is an effective treatment with which to relieve pain, deformity, restore mobility and reconstruct an elbow with near normal function when the elbow joint is damaged due to trauma or disease, such as advanced osteoarthritis and rheumatoid arthritis.^1^ TEA has previously been used to treat rheumatoid arthritis. Historically, patients treated with TEA were often in low demand, thus resulting in satisfactory efficacy with low complication rates.^2^ With the improvement of operating skills and prosthesis design, indications have expanded to include post‐traumatic instability, osteoarthritis, fracture sequelae, and acute distal humeral fractures in the elderly.^3^, ^4^ In the past, TEA was predominantly used to treat rheumatoid arthritis.^5^ However, a previous study revealed a reduced global trend in which the rate of TEAs used to treat rheumatoid arthritis reduced from 61% to 46%.^5^ This may be explained by advances in medical management, and the fact that most cases of rheumatoid arthritis are treated non‐surgically.

With the improvement of prosthesis types, selecting the correct treatment for the triceps has become the key to successful surgery. Furthermore, the concern for complications has gradually focused on the triceps after surgery. Thus, surgeons have begun to seek different surgical approaches. Techniques to deal with the triceps and exposing the elbow joint can be classified into two broad categories: triceps‐on, also known as the triceps‐preserving approach, or triceps‐off; these techniques differ based on the fact that they either preserve or compromise ulnar attachment of the triceps, respectively.^6^ Previously, the commonly reported approaches included the triceps‐off approach, which was first reported by Campbell,^7^ the Bryan–Morrey approach,^8^ which elevates the entire soft tissue envelope from a medial to a lateral direction, and the triceps‐preserving approach, which was originally developed by Pierce and Herndon.^9^ There is no consensus as to which of these approaches is the best because all of these techniques are associated with their own advantages and disadvantages.

The triceps‐off approach can provide better visualization of the joint surface but with a higher rate of complication with regard to triceps weakness and disturbance.^10^, ^11^, ^12^, ^13^ Furthermore, immobilization of the elbow for a period of time after TEA with the triceps‐off approach can compromise postoperative rehabilitation.

Over recent years, the triceps insertion reservation approach for TEA has become a hot topic of discussion due to the fact that it does not interfere with the strength of the triceps. Some surgeons have advocated that satisfactory postoperative outcome relies mostly on favorable function of triceps, otherwise, postoperative outcome can be poor.^14^ Without disturbing the extensor mechanism, immediate exercise could be undertaken for TEA with the triceps‐preserving approach, thus resulting in better clinical results and a lower rate of triceps‐related complications.^10^ Other studies have reported similar outcomes, favoring the triceps‐preserving approach.^15^, ^16^, ^17^ However, few studies have focused on the position and alignment of the implant, which might be impaired by the triceps‐preserving approach due to reduced exposure of the elbow joint.^6^, ^11^, ^18^ A previous study reported that functional outcomes are also affected by prosthetic component positioning.^19^ It is also known that implant malalignment can increase loading patterns,^20^ which might cause early loosening of the prosthesis and polyethylene wear.^21^ Whether this disadvantage of the triceps‐preserving approach will lead to implant malalignment and loosening after surgery over a long follow‐up period still needs to be clarified.

The aims of this study were: (i) to evaluate the efficacy of TEA with a triceps‐preserving approach over a relatively long period of follow‐up; (ii) to investigate the causes and prevention of TEA complications; and (iii) to compare the outcomes of TEA to treat arthropathy with that of TEA to treat acute distal humerus fracture.

## Methods

### Patients and Materials

The inclusion criteria were as follows: (i) patients with end‐stage osteoarthritis or rheumatoid arthritis of the elbow with severe pain and dysfunction; (ii) elderly patients with type C distal humerus fracture according to the Arbeitsgemeinschaft für Osteosynthesefragen (AO) classification as well as severe osteoporosis according to bone mineral density (BMD); (iii) patients who underwent primary TEA with a triceps‐preserving approach; and (iv) patients who were followed up for more than 4 years postoperatively.

The exclusion criteria were as follows: (i) pathological fracture of the distal humerus; and (ii) combined with nerve injury or triceps dysfunction before surgery.

The present study retrospectively collected clinical and radiographic outcomes of patients who underwent the TEA with triceps‐preserving approach in our hospital and was approved by the ethics committee of the local hospital (Reference: 2022‐4‐29‐1). From January 2010 to January 2018, 23 cases were retrospectively reviewed. During the study period, all TEAs were performed by the same senior surgeon with the Coonrad–Morrey semi‐constrained linked elbow arthroplasty system (Zimmer, Warsaw, IN, USA). All elbows underwent preoperative anteroposterior and lateral X‐ray and CT scans, with 3D reconstruction for further evaluation of the configuration and bone stock of the elbow.

The patients were divided into two groups according to their causes for surgery: an acute fracture group and an arthropathy group (i.e., arthritis, post‐trauma sequelae and rheumatoid arthritis).

### Surgical Technique

#### Anesthesia and Position

All operations were performed under general anesthesia with a brachial plexus block. The patients were placed in the supine position with the surgical elbow placed across the chest.

#### Approach

**Fig. 1:**
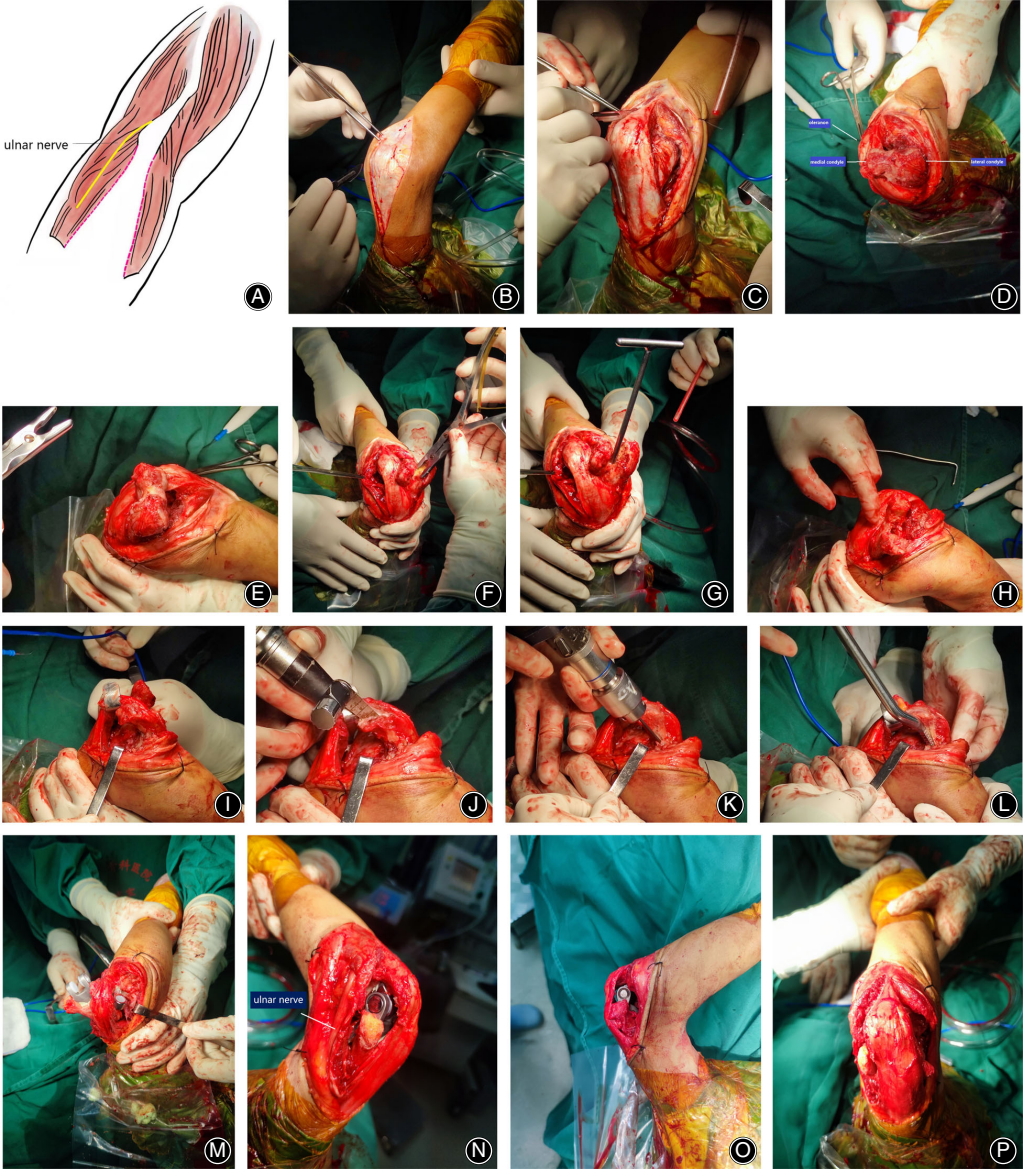
The main steps of the TEA procedure with the triceps‐preserving approach. (A) Surgical diagram: the ulnar nerve was identified and medial and lateral windows were created by elevating the triceps from the intermuscular septa and posterior humerus. (B) A midline incision was created behind the elbow. (C). Medial and lateral windows were created. (D) The medial and lateral ligaments were resected and soft tissue was released; the proximal ulna was then dislocated medially to expose the distal humerus. (E) View of the distal humerus from the lateral side. (F) Part of the distal humerus was resected. (G) The medullary canal of the humerus was broadened. (H) Osteotomy of the humerus was accomplished (I, J). Following osteotomy of the humerus, an extensive view of the proximal ulna was acquired. The tip of the olecranon was resected. (K, L) The medullary canal of the ulna was visualized and expanded. (M). The real prosthesis was inserted prior to reduction. (N) Medial view after reduction; the ulnar nerve was kept medially throughout the surgery. (O) Lateral view after reduction; the rotation center's shoulder was carefully restored. (P) The triceps was kept intact with regards to its insertion of the olecranon

A longitudinal, posterior skin incision was made just lateral to the tip of the olecranon (Fig. 1B), approximately 8 cm proximal and 6 cm distal. Full‐thickness fasciocutaneous flaps were elevated sufficiently to identify the medial and lateral edges of the triceps muscle. The ulnar nerve was then exposed and released. A medial window to the ulnohumeral joint was created by subperiosteally elevating the medial collateral ligament and flexor carpi ulnaris off the medial side of the ulna. The ulnohumeral joint was then exposed through medial and lateral windows, leaving most of the triceps insertion intact on the olecranon (Fig. 1C). The interval between the medial edge of the triceps and brachialis was identified proximally and dissected. The medial head of the triceps was elevated off the humerus to expose the medial supracondylar area.

A lateral window was generated by dissecting between the anconeus and extensor carpi ulnaris to expose the lateral supracondylar area and the posterior radiocapitellar joint (Fig. 1D,E). The triceps tendon that was inserted on the olecranon tip was maintained. The lateral collateral ligament, and a third of the extensor mass origin, were released from its origin on the lateral epicondyle. The lateral triceps was mobilized from the lateral intermuscular septum proximally.

Following complete medial and lateral mobilization, the dislocation was accomplished by hyperpronating the forearm with the proximal ulna placed medially.

### Humeral Preparation

The distal humerus preparation was often carried out before the ulna. After dislocating the proximal radius and ulna from the distal humerus, the posterior aspect of the distal humerus, the radial head, and the olecranon would be fully exposed with the additional release of the lateral and medial ligamentous structures (Fig. 1D,E).

In the arthropathy group, the trochlea and part of the capitellum were resected (Fig. 1F). Then, the medullary canal of the humerus was broadened to fit the humeral prosthesis with either condyle of the humerus kept intact (Fig. 1G). The distal portion of the humerus was resected to the level of the epicondyles (Fig. 1H).

For patients with distal humerus fractures, bone resection was not needed; thus, the fracture fragments were reduced temporarily to locate the position of the epicondyles. If the fracture line extended too proximally, then the fracture fragment would be fixed to the humerus with steel wire in order to not compromise the stability of the humeral prosthesis. Because all fractures were type C according to AO classification, no condyles were kept for the fracture group.

### Ulnar Preparation and Assembly

Following preparation, the distal humerus was turned towards the proximal ulna (Fig. 1I). With regards to the ulna, the olecranon tip was excised parallel to the longitudinal axis of the ulna to better restore the anatomic axis of the ulnar prosthesis with the forearm hyper‐pronated (Fig. 1J). The cortical and subchondral bone at the base of the coronoid were removed to expose the medullary canal of the ulna (Fig. 1K,L).

A trial reduction was performed to confirm the full range of motion of the elbow before cementing the components. The cement was injected down the medullary canal of the humerus and ulna separately and then the real prosthesis was inserted (Fig. 1M). The humeral and ulnar components were assembled and engaged by inserting the axis pin that was locked into place with a split locking ring after the cement hardened (Fig. 1N). The rotation center's shoulder was carefully restored with the central axle of the prosthesis parallel to the center of origin medial and lateral condylar (Fig. 1O). The ulnar nerve was placed *in situ* unless it was compressed prior to surgery. The ulna nerve was then pulled anteriorly (Fig. 1N) and the triceps was intact to its insertion of the olecranon throughout the surgery (Fig. 1P).

Finally, windows would be closed to cover completely the medial and lateral epicondyles and prosthesis to protect the arthroplasty from undue stress and from exposure in case of wound dehiscence. The lateral and medial collateral ligament would not be repaired. A hemovac drain was placed on the triceps fascia for the immediate postoperative period and would be removed 1–2 days after surgery.

### Postoperative Management

All patients were advised to take indomethacin for 6 weeks ask prophylaxis against heterotopic ossification. Mecobalamin was used in patients with symptoms of ulnar nerve injury. A removable brace was used to keep the elbow at 90° flexion after surgery and was removed 2 months postoperatively. Patients were managed with active flexion, extension, pronation, and supination with a full range of motion on the first postoperative day. After exercise, the elbow and prosthesis were protected by the removable splint. Three months after surgery, patients were allowed to lift 1 kg repetitively and 5 kg occasionally. As with other TEA methods, the patients were advised to limit weightlifting to 5 kg on the affected side for their lifetime.

Standard radiographs in the anteroposterior and lateral planes were performed for all patients 1–2 days after surgery to check the position of the prosthetic component.

### Postoperative Evaluations

Demographic data and indications for surgery were recorded. All patients were examined for range of motion (ROM) prior to surgery and at the last follow‐up. In the fracture group, preoperative clinical assessment could not be carried out because of pain. At every follow‐up, the pain before and after surgery was assessed by use of the visual analogue scale (VAS) score. Clinical outcomes were measured using the Mayo Elbow Performance Score (MEPS) and Disabilities of the Arm, Shoulder, and Hand (DASH) score. All patients underwent radiographic examination to evaluate prosthetic component position, alignment, and radiolucent lines. Patients were followed up (1, 2, 3, 6, and 12 months after surgery, and then yearly) regularly in the outpatient clinic. Blood loss was evaluated by measuring the blood gathered from a vacuum extractor and gauze.

#### Range of Motion (ROM)

All patients were examined for ROM of the elbow joints at the last follow‐up. Active ROM, including flexion, extension, pronation, and supination, were measured by a goniometer.

#### Visual Analogue Scale (VAS)

The VAS is the most widely used questionnaire to assess pain. Out of a total score of 10, 0 is considered as no pain, 1–3 as mild pain, 4–6 as moderate pain, and 7–9 as severe pain; a score of 10 refers to unbearable pain.

#### Mayo Elbow Performance Score (MEPS)

The MEPS is the most commonly used index to evaluate elbow function. The MEPs includes four domains: pain (45 points), stability (10 points), range of motion (20 points), and daily functional tasks (25 points). The scale ranges from 0 to 100. Scores are categorized as 90–100 = excellent, 75–89 = good, 60—4 = fair, and 0–59 = poor.

#### Disabilities of the Arm, Shoulder, and Hand Score (DASH)

The DASH score is considered reliable and valid. Thirty items are included in the DASH score to assess disability status of the upper limb. This has a total score of 100; a lower score indicates a better function in the upper limb.

#### Muscle Strength

The triceps tendon was examined for palpable gaps and muscle strength was graded as 0 to 5 according to the Medical Research Council scale. Triceps disruption was defined as an elbow extension strength of grade 2 or less. Strength was examined by performing resisted elbow extension from 90° flexion and the forearm in a neutral position. Triceps strength was graded as V (normal) if the patient was able to contract the triceps muscle normally against full resistance; IV (good), if the patient could contract against moderate resistance; III (fair), if the patient was able to extend against gravity; II (poor), if the patient could not extend against gravity with extension lag; and, I (trace) if there was just a visible contraction of muscle.

#### Radiographic Evaluation

Radiographic evaluation was performed on AP and lateral radiographs at the last follow‐up to analyze the position, alignment, and stability of the prosthesis. Radiolucent lines around the prosthesis were defined according to the Morrey classification:^22^ type 0 indicated no radiolucent line or one less than 1 millimeter wide and involving less than 50% of the interface; type I, a radiolucent line 1 millimeter wide and involving less than 50% of the interface; type II, a line more than 1 millimeter wide and involving more than 50% of the interface; type III, a line more than two millimeters wide and traversing the entire interface; and type IV, gross loosening of the implant.^22^

Flexion, extension, varus, and valgus positioning of both the humeral and ulnar components were measured in degrees, as described by Lenoir *et al*.^19^ Wearing of the bushings was also evaluated in the postoperative radiographs according to Ramsey *et al*.^23^

### Statistical Analysis

All statistical analyses were performed using SPSS software for Windows version 22 (SPSS, Chicago, IL, USA). A paired *t*‐test or Wilcoxon matched‐pairs signed‐rank test were performed to assess the difference in elbow pain and function score. A *P*‐value < 0.05 was considered to represent a significant difference.

## Results

### General Results

Twenty‐three patients were included in this study. There were seven males and 16 females. The age ranged from 46 to 85 years, with a mean age of 66.1 ± 15.33 years. The 23 patients were followed for a mean of 92.6 months (range, 52–136 months). The causes for surgery were rheumatoid arthritis in four elbows, post‐trauma sequelae in four elbows, osteoarthritis in two elbows, and acute distal humerus fracture in 13 elbows. In the arthropathy group, the course of disease ranged from 2 to 32 years, with a mean of 9.2 years. In the fracture group, the time from injury to operation was 3 to 14 days, with a mean of 5.8 days. The preoperative demographic characteristics of subgroups are shown in Table 1.

**TABLE 1:** Demographics of patients in the two groups (mean ± standard deviation)

Variables	Arthropathy group (n = 10)	Fracture group (n = 13)	*p* value	F/χ2
Sex (M/F)	3/7	4/9	0.833	0.140
Age (y)	63.7 ± 14.83 (range, 46–85)	69.3 ± 6.4 (range, 63–77)	0.077	2.227
Side (L/R)	2/8	3/10	0.913	0.476
Blood loss, mL	80.7 ± 32.8 (range, 50–150)	83.9 ± 17.5 (range, 50–100)	0.296	2.705
operative time, min	142.7 ± 44.5 (range, 110–200)	133.0 ± 32.7 (range, 105–150)	0.108	9.127
Follow‐up, month	94.6 ± 38.8 (range, 56–136)	91.5 ± 39.2 (range, 52–130)	0.772	2.025

There was no significant difference in age, gender, hand dominance, operative time, and follow‐up period between the arthropathy group and fracture group. The arthropathy groups were younger than the fracture groups, but without statistical significance (Table 1).

### Clinical Outcomes

#### Range of Motion

At the last follow‐up after the surgery, the mean flexion arc in the arthropathy group and fracture group was 100.4° ± 24.1° and 97.8° ± 28.1°, respectively. The mean pro‐supination arc in the arthropathy group and fracture group were 142.4° ± 15.2° and 139.2° ± 17.5°, respectively. There was no difference in the mean flexion arc and pro‐supination arc when compared between the two groups (Table 2).

**TABLE 2:** Postoperative outcomes of the two groups (mean ± standard deviation)

Variables	Arthropathy group (n = 10)	Fracture group (n = 13)	*P* value	F
Flexion arc, °	100.4 ± 24.1	97.8 ± 28.1	0.198	1.328
Pro‐supination arc, °	142.4 ± 15.2	139.2 ± 17.5	0.812	0.091
VAS	0.34 ± 1.7	0.31 ± 2.1	0.472	0.073
MEPS	90.8 ± 10.3	91.7 ± 17.4	0.912	1.191
DASH	37.3 ± 18.8	38.4 ± 20.1	0.763	0.012

In the arthropathy group, the flexion arc improved from 50.2° ± 29.5° preoperatively to 100.4° ± 24.1° postoperatively; this improvement was statistically significant (*P* < 0.05). The pro‐supination arc increased from 130.5° ± 19.8°preoperatively to 142.4° ± 15.2° postoperatively, but without statistical significance (*P* ≥ 0.05; Table 3).

**TABLE 3:** Clinical outcomes before and after TEA in the arthropathy group

Variables	Preoperative	Postoperative	*P* value	F
Flexion arc, °	50.2 ± 29.5	100.4 ± 24.1	0.002	0.969
Pro‐supination arc, °	130.5 ± 19.8	142.4 ± 15.2	0.087	0.708
VAS	7.5 ± 1.8	0.34 ± 1.7	0.003	0.500
MEPS	38.8 ± 22.6	90.8 ± 10.3	0.015	0.013
DASH	68.4 ± 19.2	37.3 ± 18.8	0.022	2.875

#### Visual Analogue Scale for Pain

Pain was significantly relieved in all patients after surgery. At the last follow‐up, the mean VAS score decreased significantly from 7.7 ± 2.0 (range: 5–9) preoperatively to 0.32 ± 2.1 (range: 0–2 scores) (*P* < 0.05). In the arthropathy group, VAS scores decreased from 7.5 ± 1.8 (range: 5–8 scores) to 0.34 ± 1.7 (range: 0–2 scores), and in the fracture group, VAS scores decreased from 8.0 ± 1.5 (range: 5–9 scores) to 0.31 ± 2.1 (range: 0–2 scores) (*P* < 0.05). There were no significant differences in postoperative pain when compared between the two groups at the last follow‐up (Tables 2 and 3).

#### Mayo Elbow Function Scores (MEPS)

At the last follow‐up after surgery, the mean MEPS of the arthropathy group and fracture group were 90.8 ± 10.3 points (range: 68–98 points) and 91.7 ± 17.4 (range: 76–100 points). There were no significant differences in the MEPS when compared between the two groups (*P* ≥ 0.05; Table 2). According to the MEPS, the results at the last follow‐up evaluation were excellent in 16, good in four, fair in two, and poor in one patient.

For patients with arthropathy, MEPS improved significantly from 38.8 ± 22.6 points (range: 10–50 points) before TEA to 90.8 ± 10.3 points (range: 68–98 points) postoperatively (*P* < 0.05; Table 3).

#### DASH Score

At the last follow‐up after surgery, the mean DASH in the arthropathy group and fracture group was 37.3 ± 18.8 points (range: 18–52 points) and 38.4 ± 20.1 (range: 16–60 points). There were no significant differences in the DASH when compared between the two groups (*P* ≥ 0.05; Table 2).

For patients with arthropathy, the DASH score decreased significantly from 68.4 ± 19.2 (range: 58–86 points) before surgery to 37.3 ± 18.8 points (range: 18–52 points) postoperatively (*P* < 0.05; Table 3).

#### Strength of the Triceps

At the last follow‐up, no triceps weakness occurred after TEA with normal triceps strength in 15 cases and good in eight cases. In the arthropathy group, triceps strength improved significantly after TEA (*P* < 0.05). There were no significant differences in triceps strength when compared between the two groups at the last follow‐up (*P* ≥ 0.05).

### Radiographic Outcomes

**Fig. 2:**
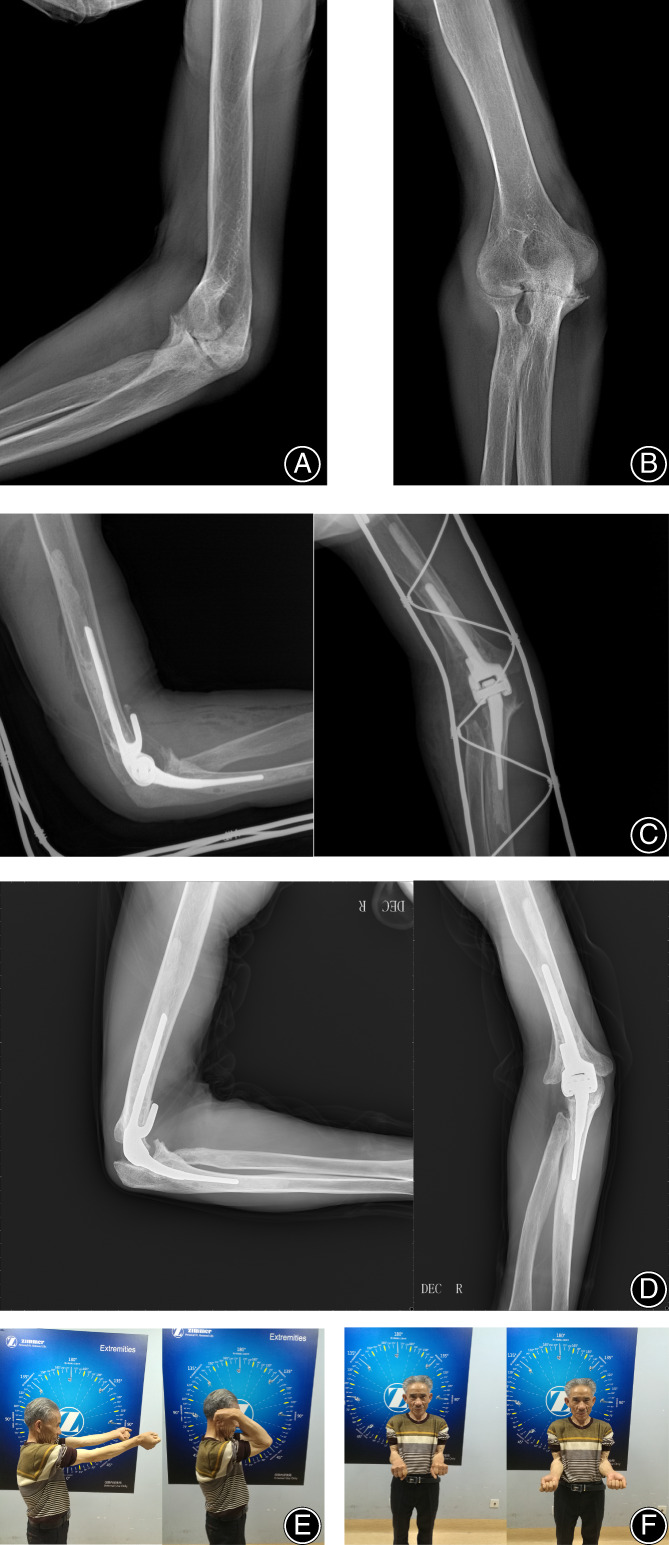
A 60‐year‐old male patient with end‐stage rheumatoid arthritis. X‐rays showed severe damage to the cartilage with a very narrow joint space (A, B). Postoperative X‐ray (C, D). At the 5‐year follow‐up after surgery, the X‐ray films of the AP view and lateral view showed that the prosthesis position was satisfactory (E, F). At the 5‐years follow‐up after surgery, the clinical outcome was satisfactory

According to the Morrey classification: three cases were found to have type 1 radiolucent lines, and the remaining 20 cases were found to have type 0 radiolucent lines. Radiolucent lines were observed around the humeral component in one area of two cases and around the ulnar component in one area of one case. No signs of loosening and malalignment were found after surgery and during follow‐up (Fig. 2).

### Complications

Two cases of heterotopic ossification were found in the fracture group after the surgery, thus causing significant restriction in the ROM of the elbow. The mean flexion arcs were 20° and 25°, respectively. The mean pro‐supination arc was 130° and 140°, respectively. Both patients refused to have surgery for release. Two cases of ulnar nerve injuries occurred during the surgery. After taking mecobalamin, one case in the arthropathy group recovered 4 months after surgery; the other patient was in the fracture group and recovered 2 years after surgery.

No weakness of the triceps muscle, infection, periprosthetic fractures, prosthesis loosening, or breakage occurred during follow‐up.

## Discussion

This study demonstrated satisfactory clinical and radiographic outcomes of TEA with a triceps‐preserving approach over a mean follow‐up of 92.6 months. At the last follow‐up, the mean mean flexion arc in the arthropathy group and the fracture group was 100.4 ± 24.1° and 97.8° ± 28.1°, respectively. At the last follow‐up, none of the patients reported pain or no pain. The MEPS in the two groups were excellent in 16 elbows, good in four elbows, fair in two elbows, and poor in one elbow. The mean DASH in the arthropathy group and fracture group were 37.3 ± 18.8 points and 38.4 ± 20.1, respectively. No weakness of the triceps strength, infection, periprosthetic fractures, prosthesis breakage was detected. We also compared the outcomes of TEA to treat arthropathy with that of TEA to treat acute distal humerus fracture. We found no differences in outcomes when compared between these two indications.

### Efficacy and Risk Factors of TEA

The primary purpose of TEA is to provide a stable, pain‐free elbow and restore the range of motion.^24^ Over recent decades, studies have reported an increasing number of TEAs performed globally.^5^ A systematic review reported the use of TEA in 9379 patients using various implant designs and surgical approaches; this study reported a mean elbow flexion angle of 129° and a mean elbow extension contracture of 30°, thus suggesting that the flexion‐extension arc approximately 90°;^25^ this was comparable to our current findings. Due to the increasing aging population and the associated likelihood of fall‐related injury, a further rise in the number of TEAs is expected. Previous studies reported that the postoperative efficacy of TEA was not as good as total hip arthroplasty or total knee arthroplasty. The outcomes of TEA differ according to indication. Better clinical outcomes were reported for TEA when used to treat rheumatoid arthritis, as well as a lower rate of complications.^26^ The initial indication of TEA is end‐stage rheumatoid arthritis of the elbow,^24^ whereas this indication has now been expanded to complex distal humeral fracture in the elderly, primary osteoarthritis, dysfunctional instability, and revision of post‐traumatic sequelae with advances in surgical techniques and implant design. More TEAs are being performed for acute distal humerus fractures (23% *vs* 38%), post‐traumatic sequelae, and primary osteoarthritis (5% *vs* 8%).^5^ Several reports have described a 10‐year survivorship following primary TEA of 85%–92% for rheumatoid arthritis, 89% for acute distal humerus fractures, 89% for primary osteoarthritis, and 42% for hemophilia;^26^ in our present study, we obtained a value of 56.5% (13/23) for acute fractures. The complication rate is reported to be 5%–30% for TEA when used to treat rheumatoid arthritis.^26^ Whereas, for other indications, such as fracture and osteoarthritis, the rate of complication is reported to be 50%.^26^ Revision rates are higher for trauma sequelae (up to 30%) when compared to rheumatoid arthritis (11–13%), acute fractures (10–11%), and primary osteoarthritis (11%).^26^

With the development of implant design, the outcomes of TEA are becoming increasingly more satisfactory. All patients in this study received the semi‐constrained prostheses. Immediate stability from the linkage mechanism can be obtained in this prosthesis and can be applied even with ligamentous insufficiency or extensive bone loss.^27^ Furthermore, in the case of elbow stiffness or deformity, this design allows for more extensive release without increasing the risk of elbow instability. Some studies have reported the 10‐year survivorship data of TEA in which the 10‐year survivorship for general indications was 91% and 85%–92% for rheumatoid arthritis, 89% for acute distal humerus fracture, 89% for osteoarthritis, 69%–80% for juvenile inflammatory arthropathy, and 42% for hemophilia.^26^ The outcomes of our study are comparable with previous reports. The results of our present study indicate that TEA is an effective way with which to treat arthropathy and acute distal humerus fracture.

Several factors have been reported to have an impact on the outcomes of TEA, such as age, sex, and body habitus. Sanchez‐Sotelo *et al*.^2^ demonstrated that male sex, concurrent traumatic pathology and the type of ulna component, all increased the risk of revision. Schoch *et al*.^28^ previously suggested caution when using TEA in younger patients under 50 years with an 82% complication rate and high rates of early mechanical failure in this subgroup. Other studies also reported that younger and more active patients, males, previous surgery, and duration of surgery, are more likely to involve mechanical failure requiring revision.^29^, ^30^ TEA with multiple previous surgeries, preoperative deformity, or ankylosis, have higher complication and revision rates.^29^, ^30^, ^31^ Patients with obesity are reported to have poor outcomes and higher rates of infection, dislocation, and periprosthetic fracture, as well as lower implant survivorship.^32^, ^33^ Furthermore, the surgeon's experience and volume, and the duration of the procedure, can influence the outcomes of TEA.^29^, ^34^, ^35^ Specialized hospitals are reported to have a lower risk of revision when compared with non‐specialized hospitals.^34^ A surgery volume of more than 10 cases per year per surgeon is reported to be associated with a better implant survival rate.^35^

The application of TEA for the treatment of acute distal humeral fractures remains controversial. However, an increasing number of TEAs are performed for acute distal humeral fracture. This poses a significant challenge for surgeons to treat osteoporotic distal humeral fracture. Considering the fact that the incidence is predicted to triple by 2030, it is especially important to identify a reasonable way to treat this fracture.^36^ Few studies have compared the outcomes of osteosynthesis with TEA. A previous study showed comparable function after TEA when compared with osteosynthesis but with a 4.4‐fold higher risk of complications for osteosynthesis.^37^ In a randomized controlled trial involving 40 patients older than 65 years, McKee *et al*. demonstrated better function after TEA over osteosynthesis at 2 years.^38^ However, other studies compared TEA with osteosynthesis, and found no difference in treatment modalities and improvement.^39^, ^40^ The outcomes of our study show that satisfactory results can be achieved when TEA is used to treat distal humerus fractures. This finding was comparable with previous reports.

### Complications of TEA

The estimated incidence of complications arising from TEA is 20% to 45%; this is much higher than that of other arthroplasties.^41^, ^42^ Infection, periprosthetic fracture, aseptic loosening, instability, implant failure, heterotopic ossification, nerve injuries, and extension weakness because of triceps insufficiency, have all been reported as complications.^12^, ^42^, ^43^ The most common indications for revision are reported to be aseptic loosening, deep infection, and periprosthetic fractures.^44^ The incidence of major complications in TEA, such as prosthesis loosening and infection, is reported to be relatively higher than that of other arthroplasties.^45^, ^46^, ^47^

Bearing surface wear is a common complication for the Coonrad–Morrey prosthesis.^29^ However, no signs of wear were found in the present cohort of patients during follow‐up; this result may be explained by the low physical demand of our patients.

Triceps weakness after TEA is relatively common and can severely impair the clinical outcomes of TEA by causing serious limitations in performing daily activities, such as pushing doors open or reaching overhead.^48^ Morrey *et al*.^49^ demonstrated an improvement of strength after TEA. The mean improvement of strength function after TEA was 92% for elbow flexion, 69% for forearm supination, 63% for forearm pronation, and 35% for hand grip strength.^49^ However, no improvement was identified in elbow extension strength after TEA. This weakness of extension strength may be explained by the fact that most posterior approaches can damage the integrity of the triceps tendon.^9^, ^49^ Another study also reported functional impairment and triceps weakness in all of their patients.^50^ Thus, the triceps must be carefully managed during surgery.

Triceps tendon rupture after TEA is a catastrophic complication for the surgeons. In a previous study, Dachs *et al*.^10^ reported seven cases of triceps rupture in 46 patients treated with TEA in a triceps off approach; four of the seven ruptures had attempted repairs and three were managed conservatively. Direct repairs were performed in all four cases; all four subsequently failed. Even worse, three of these patients developed deep infections that required multiple additional surgeries for debridement. A total of 13 additional procedures were performed on the four patients who underwent repairs.^10^ Other authors have reported similarly poor results after the attempted repair of triceps ruptures after TEA.^13^, ^51^, ^52^

The rate of major complications in our study was 8.7% (2/23) and was therefore very low. The main factor driving this low complication rate is likely to be the careful management of the triceps during surgery. This triceps‐preserving approach did have advantages without disturbing the insertion of the triceps. Antibiotics were routinely used for 3 days after surgery. Furthermore, the patients in this cohort were generally in low demand. Also, an experienced physical therapist helped the patients exercise on the first day after surgery to ensure that the rehabilitation scheme had been implemented.

### Approaches of TEA

Several approaches have been reported to deal with the triceps and expose the elbow joint during TEA. Each approach provides variation in the amount of exposure, technical difficulty, and complication profile.^18^, ^53^, ^54^ The approaches described for TEA fall into two major categories: triceps off, in which the triceps is detached from the olecranon, and triceps on or triceps‐preserving, in which most of the triceps insertion on the olecranon is kept intact.^27^

Many posterior approaches for TEA have been described in order to avoid triceps weakness and to improve extension strength.^9^, ^55^, ^56^ Campbell described a triceps longitudinal‐splitting approach in which the triceps muscle and tendon are split longitudinally from the musculotendinous junction of the triceps onto the olecranon, down to the crest of the ulna.^7^ Despite extensive injury to the triceps insertion, this approach provided limited exposure of the joint, thus resulting in a high rate of complications and a weaker extension strength than other approaches.^57^ The Bryan and Morrey approach is a widely used approach for TEA; this involves a triceps‐reflecting protocol. Some studies have revealed that this approach provides superior elbow extension strength than other approaches.^8^ This approach is reported to be superior to triceps longitudinal splitting or the triceps inverted V‐Y‐dissecting approach in terms of biomechanical properties.^58^ However, some studies have reported that the Bryan and Morrey approach has a high rate of triceps avulsion (31%); furthermore 60% of patients had less than good strength in their triceps.^9^ O'Driscoll and Morrey described a triceps‐reflecting anconeus pedicle approach, which offers more extensile exposure and preserves the neurovascular supply to the anconeus muscle.^59^ This approach preserves the anconeus, which acts as a dynamic stabilizer of the lateral elbow. However, this protocol sharply detaches the triceps insertion from the olecranon and can result in triceps in‐sufficiency in TEA.^9^

Over recent years, triceps‐preserving approaches for TEA have been gaining popularity. The triceps insertion on the olecranon is left intact, while medial and lateral windows are used. The triceps‐preserving approach allows immediate active triceps rehabilitation with better outcomes reported after TEA.^9^, ^56^ A comparative study reported a 15.2% incidence of triceps rupture in the triceps‐off group and 0% in the triceps‐on group.^10^ However, the exposed articular surface using the triceps‐preserving approach was 26%; this was almost half that of the olecranon osteotomy approach.^18^ Therefore, the triceps‐preserving approach is technically demanding, especially prosthesis insertion with proper alignment. The outcomes of this cohort reveal that the triceps‐preserving approach is effective in TEA with careful manipulation. In the current study, no cases of triceps failure were observed during the follow‐up. This might be explained by the fact that this approach does not violate the triceps attachment at the level of the proximal ulna and its periosteal attachment; this factor is believed to confer a higher risk of triceps disruption.^12^

Our results show that patients undergoing TEA with the triceps‐preserving approach can have better self‐care ability of daily living. This approach theoretically may affect the position of the prosthesis, but with the improvement of surgical techniques, it can also ensure the good positioning of the prosthesis. No loosening of the prosthesis was evident in the mid‐term follow‐up in the current study. From an intraoperative viewpoint (Fig. 1), we can conclude that extensive exposure of the elbow joint can be gained for the accurate placement of the prosthesis without compromising the position of the prosthesis. Furthermore, the center of rotation should be accurately maintained. Therefore, no signs of loosening were found during follow‐up.

### Limitations and Strengths

This study reported the clinical outcomes of TEA with a triceps‐preserving approach and explored the positioning of the prosthesis in this approach; this has not been reported previously. Our study has some limitations that need to be considered. First, this was a retrospective case series. Second, the sample size was relatively limited. TEA is not a common operation, and any single‐institution study will undoubtedly have a limited number of these patients. Further prospective and well‐designed studies are now needed with large sample sizes.

#### Conclusion

TEA with the triceps‐preserving approach can provide sufficient exposure of the articular. This is an effective technique as it significantly reduces the risk of triceps rupture, does not compromise the position of the prosthesis over a relatively long follow‐up period, and results in a satisfactory clinical and radiographic outcome with a very low rate of complications.

## Author Contributions

Qing Zhang, study design, data collection, statistics, paper writing; Ming Xiang, operation implementation; Jinsong Yang, data collection; and Fei Dai, data collection.
